# Severe rhabdomyolysis induced by bezafibrate in the treatment of severe acute pancreatitis with hyperlipidemia: A case report

**DOI:** 10.1097/MD.0000000000043009

**Published:** 2025-06-27

**Authors:** Zhengguang Geng, De Su, Bao Zhang, Fei Gao, Hua Yao, Chaojin Yang, Bao Fu, Xiaoyun Fu

**Affiliations:** a Department of Critical Care Medicine, Affiliated Hospital of Zunyi Medical University, Zunyi, Guizhou, China; b Severe Acute Pancreatitis Diagnosis and Treatment Center of Guizhou Province, Affiliated Hospital of Zunyi Medical University, Zunyi, Guizhou, China; c Department of Critical Care Medicine, Nayong Xinli Hospital, Zunyi, Guizhou, China.

**Keywords:** bezafibrate, case report, hyperlipidemia, rhabdomyolysis syndrome, severe acute pancreatitis

## Abstract

**Rationale:**

The important etiological treatment for hyperlipidemic severe acute pancreatitis is to reduce blood lipid. Statins or fibrates are often selected in clinical practice. Most reports indicate that statins or statin combination therapy can cause serious complications such as rhabdomyolysis syndrome (RM) and even poor prognosis. However, in fact, RM may also occur when fibrates alone are used to reduce blood lipid.

**Patient concerns:**

A 25-year-old man was admitted to our hospital for a 2-day history of abdominal pain, nausea, and vomiting. This patient presented with typical clinical manifestations of acute pancreatitis such as abdominal pain, abdominal distension, nausea, vomiting, and difficulty in defecation after a greasy diet. Auxiliary examinations such as blood (urine) amylase, lipase, blood lipid, abdominal computed tomography, and color Doppler ultrasound confirmed hyperlipidemic severe acute pancreatitis. However, after admission, during the treatment for reducing blood lipid, the blood creatine kinase index of the patient continued to increase, reaching more than 200 times the upper limit of normal at the highest. Accompanied by typical clinical manifestations, rhabdomyolysis was considered. After discussions among experts in the department, differentiations were mainly made from drugs after admission, recent history of toxicant exposure, history of trauma, past history of similar rhabdomyolysis, and other etiologies causing elevated myocardial enzyme spectrum. Finally, it was considered related to bezafibrate.

**Diagnoses:**

RM.

**Interventions:**

Bezafibrate tablets for lowering blood lipids were immediately discontinued. On the second day after discontinuation, there was a small decrease in creatine kinase. Then, appropriate fluid infusion, alkalinization of urine, addition of plasma exchange, and bedside continuous renal replacement therapy were administered.

**Outcomes:**

The level of muscle enzymes decreased progressively and finally returned to normal before discharge.

**Lessons:**

In clinical practice, it is necessary to dynamically monitor the changes of liver and kidney functions and myocardial enzyme spectrum when using bezafibrate tablets to treat hyperlipidemic pancreatitis.

## 1. Introduction

Rhabdomyolysis syndrome (RM) is a series of clinical syndromes caused by various reasons including trauma, drugs or poisons, genetic metabolism, and severe infections, which lead to the destruction and disintegration of striated muscles and the release of intracellular components such as creatine kinase, myoglobin, lactate dehydrogenase, and electrolytes into the extracellular fluid and bloodstream.^[1]^ The main clinical manifestations of RM include muscle pain, limb weakness, soy sauce-colored urine, fever, etc. It is often complicated by complications such as electrolyte disorders and acute renal failure. In severe cases, it can endanger life. Drugs that often cause RM mainly include lipid-lowering drugs, anti-anxiety drugs, antipsychotics, antihistamines, glucocorticoids, etc.^[2]^ For severe acute pancreatitis (SAP) with hyperlipidemia, an important etiological treatment is to lower blood lipids. Bezafibrate tablets are commonly used fibrate lipid-lowering drugs in clinical practice. Although the drug instructions clearly state that this drug has side effects such as muscle pain and rhabdomyolysis, it rarely occurs in clinical practice. Some reports are seen in combination with statin drugs. This paper retrospectively analyzes a case of RM induced during the treatment of SAP with hyperlipidemia using bezafibrate tablets. It aims to enhance the clinical understanding of RM and provide a reference for lipid-lowering treatment of acute pancreatitis with hyperlipidemia.

## 2. Case presentation

A 25-year-old man was admitted to our hospital in October 2023 for a 2-day history of abdominal pain, nausea, and vomiting. Family members reported that the patient developed upper abdominal pain and persistent abdominal distension after eating greasy food 2 days ago, mainly in the navel and upper abdominal area, reflection to the shoulders and back, no metastatic abdominal pain, accompanied by abdominal distension and profuse sweating. One day ago, nausea occurred, with vomiting of gastric contents, no hematemesis, no melena, non-projectile vomiting, no chest tightness, no chest pain, no fever, no chills, no cough, sputum production, no muscle aches, and no urine color change. He went to the local hospital and received symptomatic treatment (the specifics are unknown). Subsequently, his abdominal pain and distension worsened, and he had difficulty breathing. He was transferred to our hospital for further treatment. When the patient was admitted to the hospital, he was sent to the emergency department of our hospital and presented with the symptom of dyspnea at that time. Urgent arterial blood gas analysis was conducted (under the condition of nasal cannula oxygen inhalation at 4 L/min, with a pH of 7.32, PO_2_ of 50 mm Hg, and PCO_2_ of 32 mm Hg), and the result indicated type I respiratory failure. Meanwhile, the patient was accompanied by restlessness. In view of this, the emergency department doctor performed tracheal intubation treatment on the patient. After completing abdominal computed tomography (CT) and related physical examinations, he was diagnosed with SAP and admitted to our department. On further questioning, he had no any other notable medical history.

On admission, his temperature was 38.1 °C, respiratory rate was 22 breaths/min, heart rate was 114 beats/min, and blood pressure was 115/75 mm Hg, and body mass index was 26.5. After admission, the patient continued to be provided ventilator-assisted respiration, with a RASS score of −3. There were no jaundice, rash, or bleeding spots on the skin and mucosa of the whole body, without thyroid enlargement, carotid bruits, or cervical lymphadenopathy. The breath sounds in both lungs were clear, wet rales can be heard in both lungs, the heart boundary was normal, the heart rhythm was regular, and no pathological murmur could be heard in the auscultation area of each valve. Physical examination showed a bulging abdomen with tenderness in the epigastrium, no gastrointestinal type and peristaltic wave, no varicose veins in the abdominal wall, no rebound tenderness, and no palpable liver and spleen. The rest of the physical examination was roughly normal.

**Figure 1.**
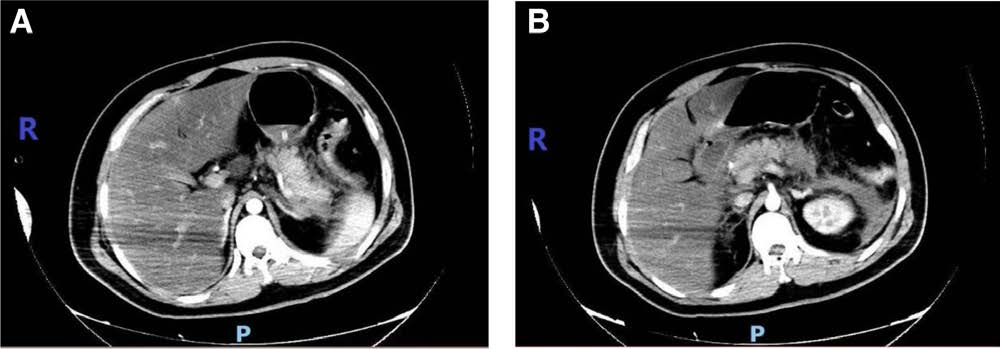
Enhanced CT of the upper abdomen: pancreatic edema, necrotic exudation around the pancreas and around the left kidney, fatty liver or hepatic edema. CT = computed tomography.

Laboratory examinations on admission, including routine blood, electrolytes, renal function, amylase, cardiac enzymes, plasma lipids, etc, are shown in Table 1 below. T helper 1 and T helper 2 cytokines detection: interleukin (IL)-6 level was 632.43 pg/mL (normal range 1.18–5.30 pg/mL), while the levels of IL-2, IL-4, IL-6, IL-10, tumor necrosis factor-α, and interferon-γ were all within normal ranges. Liver function and coagulation function showed no obvious abnormalities. Blood gas analysis showed (FiO_2_ 60%): pH of 7.41, PaO_2_ of 79.1 mm Hg, and PCO_2_ of 27.7 mm Hg. Chest CT at admission showed double lung pneumonia. CT enhancement of the upper abdomen (Fig. 1): acute pancreatitis, peritonitis, abdominal effusion, thickening and edema of the duodenal wall, fatty liver or hepatic edema. Abdominal ultrasound showed fatty liver and suggestive of pancreatitis. Electrocardiography indicated sinus tachycardia.

**Table 1:** Relevant test indicators of the patient.

Test indicators	Detected value	Normal range
Blood routine		
Total white blood cell count (×10^9^/L)	14.17	3.5–9.5
Percentage of neutrophils	0.82	0.40–0.75
Total red blood cell count (×10^12^/L)	5.72	4.3–5.8
Hemoglobin (g/L)	171.0	130–175
Hematocrit	0.52	0.4–0.5
Total platelet count (×10^9^/L)	342	100–300
D-dimer (μg/mL)	2.03	<0.5
Calcium (mmol/L)	1.82	2.2–2.7
Renal function		
Blood urea nitrogen (mmol/L)	10.5	2.8–7.2
Creatinine (μmol/L)	217	41–109
Creatinine clearance rate (ml/(min 1.73 m^2^))	42.79	85–125
Blood amylase (U/L)	683	0–220
Urine amylase (U/L)	2350	0–1200
Lipase (U/L)	839.78	0–60
Myocardial enzymes		
Creatine kinase (U/L)	509	38–174
Creatine kinase isoenzyme (U/L)	22	0–24
Lactate dehydrogenase (U/L)	530	140–271
α-Hydroxybutyrate dehydrogenase (U/L)	288	90–180
Myoglobin (ng/mL)	336	28–72
High-sensitivity troponin T (ng/L)	8.12	<14.00
Blood lipids		
Triglyceride (mmol/L)	20.95	<1.7
Total cholesterol (mmol/L)	13.01	<5.2
High-density lipoprotein cholesterol (mmol/L)	3.41	0.91–1.55
Low-density lipoprotein cholesterol (mmol/L)	0.77	<4.1
High-sensitivity C-reactive protein (mg/L)	181.511	0.068–8.200
Procalcitonin (ng/mL)	0.91	<0.50

**Figure 2.**
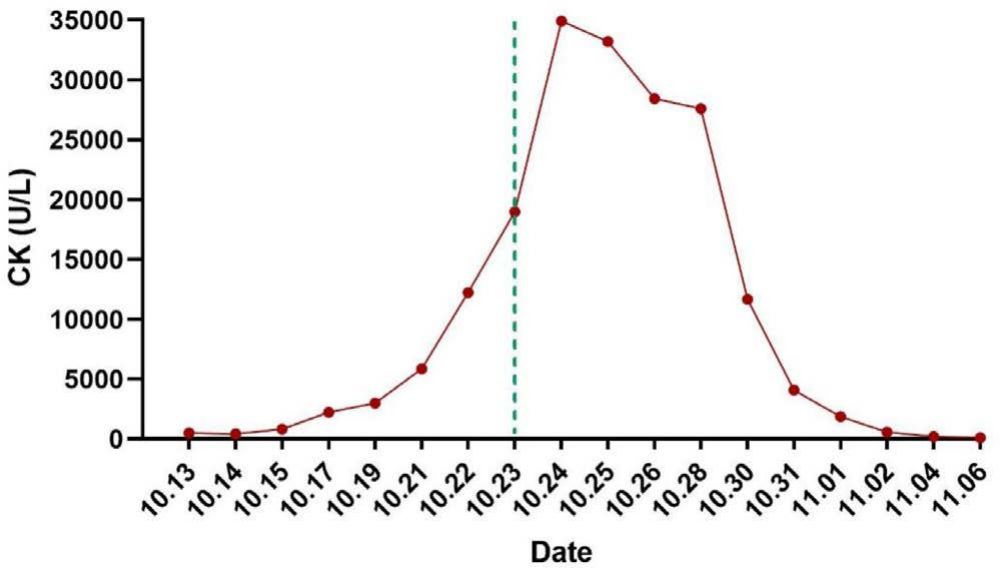
Changes in CK after admission. The dotted line indicates the time when bezafibrate tablets were discontinued. CK = creatine kinase.

Preliminary diagnoses based on the present and past history, clinical manifestations, physical examination, and comprehensive auxiliary examinations, are as follows: (1) severe acute pancreatitis (hyperlipidemia type); (2) acute respiratory distress syndrome; (3) bilateral pneumonia; (4) acute kidney injury; (5) hyperlipidemia; (6) electrolyte imbalance (hypocalcemia). Intensive care treatment, assisted ventilation with a ventilator, infection control, gastric acid protection, anti-inflammatory treatment, inhibition of pancreatic enzyme secretion, promotion of bowel movements, reduction of blood lipids (heparin), appropriate fluid resuscitation, nutritional support, and maintenance of fluid and electrolyte balance. On the second day of admission (14th October), an additional lipid-lowering plan was added to the existing treatment regimen: Bezafibrate tablets 0.4 g to be administered via gastric tube 3 times a day, while the rest of the treatment plan remains unchanged. During the treatment process, monitoring of the patient’s creatine kinase (CK) levels gradually increased, accompanied by progressive deterioration of liver and kidney function. The patient gradually developed oliguria, dark urine, anuria, and underwent comprehensive venous blood pathogen metagenomic sequencing (DNA + RNA), thyroid function, anti-nuclear antibodies, anti-nuclear anti-body spectrum, complement levels, anti-phospholipid antibody spectrum, hepatitis B surface antigen, hepatitis A virus, hepatitis E virus, Epstein–Barr virus, cytomegalovirus, all showing no obvious abnormalities. Following departmental discussion, it was considered that the patient’s rhabdomyolysis was caused by bezafibrate tablets. The bezafibrate tablets were discontinued on October 23rd, and the patient received appropriate fluid replacement, urine alkalization, plasma exchange therapy, and bedside hemofiltration treatment. The levels of muscle enzymes showed a progressive decline (Fig. 2), and upon discharge, the patient’s CK had returned to normal levels.

## 3. Discussion

This patient presented with typical clinical manifestations of acute pancreatitis such as abdominal pain, abdominal distension, nausea, vomiting, and difficulty in defecation after a greasy diet. Auxiliary examinations such as blood (urine) amylase, lipase, abdominal CT, and color Doppler ultrasound confirmed acute pancreatitis. Moreover, acute respiratory distress syndrome and acute kidney injury occurred within a short period of time. Therefore, the diagnosis of SAP is definite. Acute pancreatitis commonly has causes such as biliary, hyperlipidemic, alcoholic, drug-induced or toxic, iatrogenic, and traumatic factors.^[3]^ Upon admission, routine abdominal CT and abdominal ultrasound showed no obvious gallstones, bile duct dilatation, or bile stasis. Liver function was normal, with no history of alcohol consumption, trauma, medication, or toxin exposure prior to illness. Blood lipid levels upon admission were ten times higher than the upper limit of normal. Both abdominal CT and ultrasound indicated a clear diagnosis of fatty liver, hence suggesting SAP of the hyperlipidemic type. The most commonly used lipid-lowering drugs for hyperlipidemic acute pancreatitis are statins and fibrate drugs. This paper reports a case of RM induced by the use of bezafibrate tablets in lipid-lowering treatment for SAP of hyperlipidemia type. However, what is the cause of RM and what is the mechanism by which bezafibrate causes rhabdomyolysis? It is hoped that this paper can provide warnings and reminders for lipid-lowering treatment of SAP of hyperlipidemia type in clinical practice, so as to avoid or reduce severe rhabdomyolysis caused by this drug, reduce renal injury in patients, shorten the hospital stay of patients with SAP, and reduce the economic burden.

### 3.1. Etiologies, diagnosis, and differential diagnosis of rhabdomyolysis

RM refers to a clinical syndrome of rhabdomyolysis caused by a series of factors affecting the cell membrane, membrane channels, and energy supply of striated muscles. Currently, there is no unified diagnostic standard for RM internationally. Elevated CK and myoglobin are important bases for diagnosing rhabdomyolysis. CK usually rises within 12 hours after muscle injury, reaches a peak within 24 to 72 hours, and returns to normal within about 5 days after muscle injury stops.^[4]^ For the diagnosis of this disease, clinical consensus mostly recommends judging from the predisposing factors (trauma, drugs, poisoning, metabolism, etc) causing rhabdomyolysis, clinical manifestations (myalgia, weakness, fever, oliguria, soy sauce-colored urine, etc), and auxiliary examinations (electrolyte disorders, increased myocardial enzyme spectrum, etc). Torres PA et al believe that CK is the most sensitive laboratory test for evaluating possible rhabdomyolysis.^[5,6]^ The gold standard for laboratory diagnosis is the determination of plasma CK.^[7]^ The serum CK level of this patient was significantly increased and more than ten times the upper limit of normal. Combined with the increase of LDH, α-hydroxybutyrate dehydrogenase, and myoglobin, and soy sauce-colored urine, the diagnosis of rhabdomyolysis in the patient is definite. Tracing the myocardial enzyme spectrum of the patient in the local hospital before hospitalization, CK was also slightly increased (<5 times the upper limit of normal). However, after admission, CK gradually increased, reaching more than 200 times the upper limit of normal at most. The etiologies are mainly differentiated from drugs after admission, recent history of toxicant exposure, history of trauma, previous history of similar rhabdomyolysis, and other etiologies that cause an increase in myocardial enzyme spectrum (hypothyroidism or hyperthyroidism, autoimmune diseases such as systemic lupus erythematosus, myocardial infarction, etc).

The pathogenic factors of RM include genetics, muscle trauma, infection, drugs, toxins, and other severe diseases that can cause energy imbalance in the body. Among them, drugs and toxins are the main etiologies. After admission, this patient was treated with cefoperazone sodium and sulbactam sodium for anti-infection, ulinastatin sodium for anti-inflammation, octreotide acetate injection for inhibiting pancreatic enzyme secretion, omeprazole sodium for inhibiting gastric acid secretion and protecting gastric mucosa, and heparin combined with bezafibrate tablets for lowering blood lipids. (1) After admission, the patient’s medical history was repeatedly inquired. Neither the patient nor their parents and siblings had symptoms similar to rhabdomyolysis. There was no history of trauma, no surgeries had been performed, and there was no intake of special drugs, toxins, or alcohol before the illness. Thus, rhabdomyolysis caused by genetic, traumatic, and toxic factors can be excluded. (2) After admission, the thyroid function tests were completed and no obvious abnormalities were found. Thus, rhabdomyolysis caused by hyperthyroidism or hypothyroidism can be excluded. (3) Tests such as antinuclear antibody, antinuclear antibody spectrum, quantitative immunoglobulin, complement level, and antiphospholipid antibody spectrum were completed and no obvious abnormalities were found. Thus, changes in myocardial enzyme spectrum related to autoimmune diseases can be excluded. (4) Venous blood pathogen metagenomic sequencing (DNA + RNA), hepatitis B virus, hepatitis A virus, hepatitis E virus, Epstein–Barr virus, cytomegalovirus, etc were all negative. Thus, rhabdomyolysis caused by severe infection can be excluded. (5) The bedside electrocardiogram suggested sinus tachycardia. The echocardiogram suggested no obvious abnormalities in the left and right heart chambers. Troponin T was within the normal range, and CK-MB was slightly increased (during the entire course of the disease). Diseases that need to be differentiated such as myocardial infarction and cardiomyopathy can be excluded.

Considering that the patient’s CK level continued to rise after admission, accompanied by clinical manifestations such as deteriorating renal function, oliguria, and soy sauce-colored urine. After departmental discussion and exclusion of the above etiologies, the treatment medications since admission were investigated one by one. Except for bezafibrate tablets, which have been reported in a small number of cases to cause rhabdomyolysis, the other drugs have no relevant adverse risks. Then, bezafibrate tablets for lowering blood lipids were immediately discontinued. On the second day after discontinuation, there was a small decrease in CK. Then, appropriate fluid infusion, alkalinization of urine, addition of plasma exchange and bedside continuous renal replacement therapy (CRRT) were administered. The level of muscle enzymes decreased progressively and finally returned to normal before discharge.

### 3.2. Possible mechanism of rhabdomyolysis induced by bezafibrate

Bezafibrate is a derivative of chlorobutanol acid used as a lipid-regulating drug. It is commonly used clinically to treat hypertriglyceridemia, hypercholesterolemia, and mixed hyperlipidemia, with a stronger effect on reducing triglycerides than cholesterol. Common side effects include indigestion, nausea, vomiting, abnormal liver function, as well as myositis, muscle pain, and rhabdomyolysis. Despite this adverse reaction, previous clinical reports have shown that the incidence of rhabdomyolysis with fibrates and statins as single drug therapy is very low (0.0028–0.0096%). It is only when these drugs are used in combination that the incidence increases to 0.015–0.021%.^[8]^ The specific mechanism by which bezafibrate induces rhabdomyolysis in hyperlipidemic pancreatitis is not yet clear. It may be related to the following factors: (1) Fibrates bind to proliferator-activated receptor alpha in peroxisomes, subsequently stimulating fatty acid oxidation and reducing hepatic lipid synthesis rate, not only lowering triglycerides but also inhibiting cholesterol synthesis, thereby destabilizing skeletal muscle cell membranes and leading to muscle cell damage.^[9]^ (2) Inhibiting Cl^-^ channels hinders the depolarization of muscle cell membranes, resulting in sustained muscle contraction and promoting muscle cell damage.^[10,11]^ (3) Direct toxic effects on skeletal muscles, especially when used in combination with statins, are more pronounced. This is because both inhibit the activity of cytochrome oxidase, leading to a slower drug elimination rate.^[12]^ Additionally, their combined use inhibits the biosynthesis of geranylgeranyl pyrophosphate (GGPP) and the activation of the Rho pathway, enhances p27 expression, induces cell cycle arrest in G1 phase and cell apoptosis, resulting in the death of myoblasts and myotubes.^[8]^ (4) Impact on the energy metabolism of skeletal muscle cells. Skeletal muscle is the primary site for the oxidation of fatty acids and glucose, accounting for about half of body weight. Factors that lead to changes in glycolysis, glycogen breakdown, fat oxidation, and mitochondrial respiration can impair skeletal muscle function, resulting in severe myopathies and rhabdomyolysis.^[13]^ Various studies have shown that lipid-lowering medications interfere with several pathways of muscle cell energy metabolism and affect muscle lipoprotein lipase activity.^[11]^ Childhood rhabdomyolysis has a relatively common genetic origin in mutations of the LPIN1 gene. Under conditions of nutrient deficiency, lipin1 translocates to the cell nucleus and interacts with coactivators involved in metabolic gene expression, including peroxisome proliferator-activated receptor gamma coactivator 1-alpha (PGC1α), peroxisome proliferator-activated receptors alpha and gamma (PPARα, PPARγ), to regulate skeletal muscle function.^[14]^ However, bezafibrate competitively binds to PPARα, leading to dysfunction of lipin1, causing endoplasmic reticulum stress, unfolded protein response, lipid droplet accumulation, and rhabdomyolysis.

The prognosis of RM depends largely on the underlying causes of the onset and the complications.^[2]^ Since fibrate drugs are mainly excreted through the kidneys, in clinical practice, cases of rhabdomyolysis mostly occur in patients with renal insufficiency, especially those with chronic kidney disease. This case involves a patient with severe hypertriglyceridemic acute pancreatitis. During the process of using bezafibrate tablets to lower blood lipids, the patient showed a continuous increase in CK, accompanied by clinical manifestations such as oliguria, soy sauce-colored urine, and impairment of renal function. Through clinical differentiation, it was considered that rhabdomyolysis might be caused by bezafibrate tablets. After discontinuing the drug and receiving treatments such as CRRT, the patient’s urine output and myocardial enzyme spectrum gradually improved. Eventually, the CK level decreased to the normal range, and the patient was discharged in improved condition. The patient’s renal function impairment is acute kidney injury caused by rhabdomyolysis induced by the drug, which is a complication of rhabdomyolysis rather than a basic disease based on chronic renal insufficiency. Similar to the good prognosis of this patient, most reports indicate that acute renal function impairment caused by bezafibrate tablets is completely reversible.^[15]^ In general, if rhabdomyolysis can be detected early and treated promptly, it often has a positive prognosis.

## 4. Conclusion

This study hopes to be helpful for the clinical diagnosis and treatment of related cases. When encountering hyperlipidemic pancreatitis and using bezafibrate to lower blood lipids, it is necessary to dynamically detect clinical test indicators such as liver and kidney function and myocardial enzymes. When symptoms such as muscle pain, weakness, oliguria, and even soy sauce-colored urine appear, one should be vigilant about rhabdomyolysis and need to stop the drug in time to achieve early detection and early treatment.

## Author contributions

**Conceptualization:** Zhengguang Geng, Bao Fu, Xiaoyun Fu.

**Data curation:** De Su, Bao Zhang.

**Visualization:** Fei Gao, Hua Yao, Chaojin Yang.

**Writing – original draft:** Zhengguang Geng, De Su, Bao Zhang.

**Writing – review & editing:** Bao Fu, Xiaoyun Fu.
